# Deep Learning-Based Parkinson’s Disease Classification Using RGB Plantar Pressure Gait Images: A Comparative Study of CNN and Transformer Architectures

**DOI:** 10.3390/diagnostics16183021

**Published:** 2026-09-17

**Authors:** Chun-Yu Li, Yu-Wen Hung, Jia-Lang Xu

**Affiliations:** 1 Department of Life Sciences, College of Life Sciences, National Chung-Hsing University, Taichung 402, Taiwan; 2 Adult Respiratory Therapy Division, Changhua Christian Hospital, Changhua 500, Taiwan; 3 Department of Applied Statistics, National Taichung University of Science and Technology, Taichung 404, Taiwan

**Keywords:** Parkinson’s disease, plantar pressure, gait analysis, vertical ground reaction force, deep learning, signal-to-image representation

## Abstract

**Background/Objectives**: Parkinson’s disease (PD) is associated with gait abnormalities that provide quantitative information on motor function. This study developed and evaluated a signal-to-image deep learning framework for distinguishing participants with diagnosed PD from healthy controls using plantar pressure gait signals. **Methods**: VGRF signals from the PhysioNet Gait in Parkinson’s Disease Database were transformed into RGB images encoding left-foot pressure, right-foot pressure, and the absolute bilateral difference. ResNet50, EfficientNet-B0, ViT-Tiny, and Swin-Tiny were evaluated at three resolutions and compared with 1D-CNN and BiLSTM baselines. Repeated subject-level five-fold cross-validation with three repetitions and participant-level bootstrap analysis were performed. **Results**: Among RGB models, ViT-Tiny at 384 × 384 achieved a mean accuracy of 77.64%, F1-score of 82.82%, and AUC of 86.40%. In the Swin-Tiny 384 × 384 ablation, the absolute bilateral-difference representation achieved the highest mean AUC (86.68%). However, its participant-level AUC advantage over RGB was not statistically conclusive (ΔAUC = 0.030, 95% CI: −0.011 to 0.068). **Conclusions**: Signal-derived gait images provide a feasible approach for PD classification, with bilateral-difference information showing potential discriminative value.

## 1. Introduction

Parkinson’s disease (PD) is a common and progressive neurodegenerative disorder characterized by the progressive loss of dopaminergic neurons in the substantia nigra, resulting in the cardinal motor symptoms of bradykinesia, rigidity, resting tremor, and postural instability [1]. Among these manifestations, gait impairment is one of the most prominent clinical features and represents a major contributor to falls, disability, and reduced quality of life. Compared with healthy individuals, patients with PD exhibit distinct gait abnormalities, including reduced stride length, decreased gait speed, increased cadence accompanied by shorter step length, shuffling gait, diminished heel strike, prolonged double-support time, and increased gait variability [2,3]. These alterations in gait reflect the progressive impairment of motor control and have been widely recognized as objective biomarkers for the early detection, disease monitoring, and personalized management of PD [4]. Quantitative gait analysis has emerged as an important approach for objectively evaluating disease severity and supporting clinical decision-making.

Conventional assessment of gait and motor impairments in patients with Parkinson’s disease (PD) primarily relies on neurologists’ clinical observations and standardized rating scales during outpatient visits. However, these assessments are inherently subjective and may not accurately reflect patients’ motor performance in daily life, highlighting the need for objective and continuous gait evaluation methods [5]. Consequently, quantitative gait analysis has emerged as an important approach for the early detection and monitoring of PD. Previous studies have demonstrated that gait parameters can effectively distinguish patients with early-stage PD from healthy individuals and provide valuable information for assessing disease severity [6]. Recent advances in computer vision, wearable sensing technologies, and artificial intelligence have further promoted the development of objective gait assessment. Vision-based gait analysis combined with deep learning enables the extraction of three-dimensional skeletal keypoints from gait videos, providing a low-cost, non-contact, and objective solution for neurological disorder assessment [7]. Quantitative spatiotemporal gait parameters, including stride length, step length, stance duration, swing time, gait speed, and double-support time, have been recognized as reliable biomarkers for the early detection and severity assessment of PD. Machine learning models trained on these gait features have demonstrated excellent diagnostic performance, highlighting the clinical value of objective gait analysis [8]. Furthermore, gait and postural transition parameters derived from wearable sensors have shown promising performance in differentiating patients with early-stage PD from those with essential tremor, supporting the potential of quantitative gait analysis for early diagnosis and clinical decision-making [9]. Among gait abnormalities, freezing of gait (FoG) is one of the most disabling motor symptoms of PD and is strongly associated with impaired mobility, reduced quality of life, and an increased risk of falls. Recent studies have demonstrated that deep learning models can automatically learn discriminative gait representations from sensor data and accurately detect or even predict FoG episodes in real time, outperforming conventional approaches based on handcrafted gait features [10,11]. Despite these encouraging findings, the generalizability of existing deep learning models across different datasets and clinical environments remains limited, highlighting the need for more robust and standardized artificial intelligence approaches for automated gait assessment [12]. Beyond gait analysis, artificial intelligence has demonstrated substantial potential for supporting clinical decision-making across a wide range of neurological and medical applications. Machine learning models have successfully integrated heterogeneous clinical, imaging, and physiological data to improve disease classification, risk stratification, and personalized patient management, providing a strong foundation for intelligent clinical decision support systems (CDSSs) [13]. The integration of edge computing with artificial intelligence has enabled real-time clinical prediction with reduced computational burden, improved system stability, and enhanced deployment efficiency in healthcare environments [14]. The successful translation of deep learning models into routine clinical practice depends not only on predictive accuracy but also on robust generalization across independent datasets and diverse clinical scenarios. Recent studies have highlighted that many deep learning models exhibit substantial performance degradation when evaluated on external datasets, emphasizing the importance of systematically assessing model robustness and generalizability for reliable clinical deployment [15].

The objective of this study was to develop and systematically evaluate a signal-to-image deep learning framework for distinguishing participants with diagnosed Parkinson’s disease (PD) from healthy controls using plantar pressure gait data. The proposed framework transforms bilateral vertical ground reaction force (VGRF) signals into standardized RGB gait images, in which left-foot pressure, right-foot pressure, and the absolute bilateral difference |L − R| are encoded into separate image channels. This representation enables signal-derived gait data to be processed using established image-based deep learning architecture without requiring manual extraction of predefined gait features. To evaluate the proposed framework, four representative CNN- and transformer-based architectures, including ResNet50, EfficientNet-B0, Vision Transformer (ViT-Tiny), and Swin Transformer (Swin-Tiny), were evaluated at three input resolutions (224 × 224, 384 × 384, and 512 × 512). Direct time-series models, including 1D-CNN and BiLSTM, were additionally evaluated as reference baselines. Representation ablation experiments using R, G, RG, RGB, and B encodings were conducted with Swin-Tiny at 384 × 384 to examine the contribution of unilateral, bilateral, and absolute bilateral-difference information. Model performance was assessed using repeated stratified subject-level five-fold cross-validation with three repetitions. Fold-level results were used to summarize performance variability, while aggregated participant-level out-of-fold predictions and participant-level bootstrap resampling were used to quantify uncertainty and compare selected configurations. Computational efficiency was also evaluated. Overall, this study investigated how signal representation, network architecture, and input resolution influence discrimination between diagnosed PD and healthy controls within the evaluated dataset.

The main contributions of this study are summarized as follows:

1. Signal-to-image representation: A standardized RGB gait-image encoding framework was developed to transform bilateral VGRF signals into image-compatible representations incorporating left-foot pressure, right-foot pressure, and the absolute bilateral difference |L − R|.
2. Model and resolution evaluation: Four CNN- and transformer-based architectures were systematically evaluated at three input resolutions and compared with two direct VGRF time-series baselines under a consistent subject-level evaluation protocol.
3. Controlled representation ablation: R, G, RG, RGB, and B representations were evaluated using a fixed Swin-Tiny architecture at 384 × 384 resolution to examine the contribution of unilateral, bilateral, and absolute bilateral-difference information while avoiding confounding between representation and architecture within the ablation analysis.
4. Robust subject-level evaluation: Repeated stratified subject-level five-fold cross-validation with three repetitions was employed to prevent participant leakage and reduce dependence on a single data partition. Participant-level out-of-fold predictions were further aggregated to provide one prediction per participant.
5. Participant-level statistical and computational assessment: Uncertainty and selected between-model differences were evaluated using participant-level bootstrap resampling with 2000 iterations and 95% confidence intervals, together with computational efficiency measures, providing an evaluation beyond classification accuracy alone.

## 2. Related Work

Deep learning has become a major approach for modern gait recognition because of its ability to automatically learn discriminative spatial and temporal representations from diverse gait data, reducing the dependence on handcrafted feature extraction [16]. Among these approaches, convolutional neural networks (CNNs) have been widely applied to image-based gait recognition and clinical gait assessment, demonstrating the ability to extract informative representations from gait images and distinguish normal and pathological movement patterns [17,18]. Advances in computer vision have further enabled non-contact gait assessment through three-dimensional skeletal keypoints and spatial–temporal modeling [19]. More recently, transformer-based architectures have extended these capabilities by using self-attention to capture long-range dependencies within gait representations. Vision Transformers (ViTs) have demonstrated competitive performance in gait recognition [20], while model-based approaches combining skeleton representations, graph convolutional networks, and generative learning have improved robustness to variations such as viewing angle [21]. Collectively, these developments demonstrate the potential of deep learning to integrate spatial, temporal, and structural information for objective gait analysis and neurological disorder assessment. Similar advances in medical image analysis further support the capability of deep neural networks to extract clinically relevant patterns from image-based representations [22]. Image-based deep learning has also been investigated specifically for Parkinson’s disease (PD) classification. CNN-based analysis of offline handwriting images has achieved high classification performance across public datasets including HandPD, NewHandPD, PaHaW, and UCI, with reported accuracies of 94.74% on NewHandPD and 100% on the smaller UCI dataset [23]. More recently, a ViT-based approach applied to hand-drawn Archimedean spiral images achieved 93.09% accuracy when evaluated on the independent NewHandPD dataset, indicating that self-attention-based architectures can also capture discriminative PD-related motor patterns from static images [24]. These image-based studies have primarily focused on handwriting and therefore characterize upper-limb motor manifestations.

## 3. Materials and Methods

**Figure 1:**
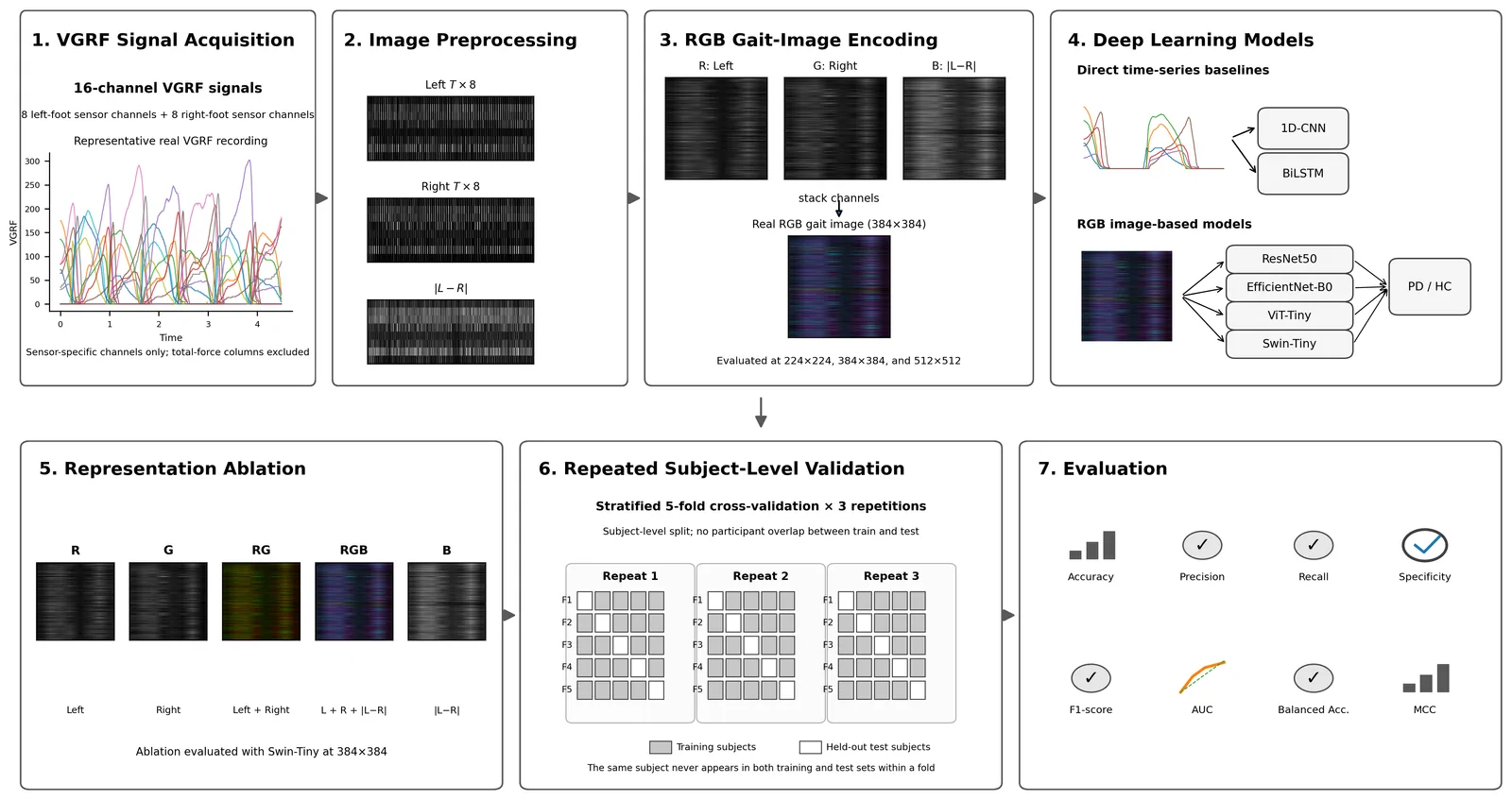
Overview of the Proposed VGRF-to-Image Classification Framework. Real VGRF signals and signal-derived matrices from one representative recording are shown for illustration.

Figure 1 illustrates the overall workflow of the proposed Parkinson’s disease classification framework based on signal-derived RGB gait images. The framework consists of seven stages: VGRF signal acquisition, image preprocessing, RGB gait-image encoding, deep learning model evaluation, representation ablation, repeated subject-level validation, and performance evaluation. Vertical ground reaction force (VGRF) signals from 16 sensor-specific channels, comprising eight channels from each foot, are extracted from each complete gait recording. The 16 sensor channels are then separated into left-foot and right-foot matrices, and the absolute bilateral difference is calculated as |L − R|. For image construction, the left-foot, right-foot, and bilateral-difference matrices are independently normalized within each recording using Min–Max normalization and directly resized using bilinear interpolation. No temporal segmentation, padding, truncation, or gait-cycle normalization is applied. The normalized left-foot, right-foot, and absolute bilateral-difference matrices are encoded into the red, green, and blue channels, respectively, to generate RGB gait images. The images are evaluated at three input resolutions: 224 × 224, 384 × 384, and 512 × 512. Four image-based deep learning architectures, including ResNet50, EfficientNet-B0, ViT-Tiny, and Swin-Tiny, are evaluated. In parallel, 1D-CNN and BiLSTM models are included as direct time-series baselines using the 16-channel VGRF sequences. To examine the contribution of different signal representations, an ablation analysis is conducted using R, G, RG, RGB, and B encodings with Swin-Tiny at 384 × 384 resolution. All models are evaluated using repeated stratified subject-level five-fold cross-validation with three repetitions, ensuring that recordings from the same participant do not appear in both training and test sets within a fold. Model performance is assessed using accuracy, precision, recall, specificity, F1-score, AUC, balanced accuracy, and MCC. For participant-level evaluation, out-of-fold probabilities are aggregated to one prediction per participant, and 2000 participant-level bootstrap resamples are used to estimate 95% confidence intervals.

### 3.1. Data Collection

The gait dataset used in this study was obtained from the publicly available PhysioNet Gait in Parkinson’s Disease Database [25]. The original cohort included 93 individuals with idiopathic Parkinson’s disease (PD) and 73 healthy controls (HCs). One HC was excluded because no corresponding gait recording was available, yielding a final analytical cohort of 165 participants (93 PD and 72 HCs) with 306 gait recordings. The mean age of the PD and HC groups was 66.3 years, and males represented 63% and 55% of the respective groups. During data acquisition, participants walked continuously at their self-selected comfortable pace on a level surface for approximately two minutes. Vertical ground reaction force (VGRF) signals were recorded using an in-shoe plantar pressure measurement system (Ultraflex Computer Dyno Graphy, Infotronic Inc., Vriezenveen, NL, USA). Eight force sensors were positioned beneath each foot, resulting in a total of sixteen plantar pressure sensors. Force signals were sampled at 100 Hz, generating continuous plantar pressure time-series data throughout the walking trials.

### 3.2. Data Preprocessing

The raw plantar pressure data were obtained from the PhysioNet Parkinson’s Disease Gait Database. Each walking trial consisted of continuous vertical ground reaction force (VGRF) signals recorded at 100 Hz from 16 plantar pressure sensors, with eight sensors positioned beneath each foot. After removal of the time column, the first eight VGRF channels were assigned to the left-foot matrix $L\in R^{T\times 8}$ and the remaining eight channels were assigned to the right-foot matrix $R\in R^{T\times 8}$, where T denotes the number of temporal samples in an individual walking trial. The rows of each matrix represented successive temporal samples, whereas the columns represented the eight ordered sensor indices. Each complete walking trial was processed as a single sample without temporal segmentation or gait-cycle extraction. The recordings were not divided into fixed-length windows; no segment length, overlap ratio, or gait-cycle normalization was applied, and no segmented windows were treated as independent samples. Missing or invalid values were removed during data preprocessing before construction of the signal-derived images.

The eight left-foot and eight right-foot channels were paired according to their corresponding sensor indices (sensor 1 with sensor 1 through sensor 8 with sensor 8). To characterize bilateral differences in plantar loading, an element-wise absolute difference matrix was calculated as:

(1) D=|L−R|

where $D\in R^{T\times 8}$ represents the bilateral-difference matrix. Because the left- and right-foot sensor signals were recorded simultaneously within the same walking trial, corresponding rows represented the same temporal samples; therefore, no additional temporal synchronization procedure was required.

For each walking trial, the left-foot, right-foot, and bilateral-difference matrices were independently normalized using matrix-level Min–Max normalization:

(2) $$\tilde{M}=\frac{M-min(M)}{\max\left(M\right)-\min\left(M\right)+E},\;M\in \{L,R,D\}$$

where M denotes the original left-foot, right-foot, or bilateral-difference matrix, min(M) and $\max\left(M\right)$ denote the minimum and maximum values calculated exclusively from the corresponding matrix within the same walking trial, $\tilde{M}$ denotes the normalized matrix, and $E=10^{-8}$ was included for numerical stability. Importantly, the normalization parameters were calculated independently for each walking trial and were not derived from the complete dataset or shared across participants or cross-validation folds. Therefore, information from validation or test subjects was not used to determine the normalization parameters, thereby preventing normalization-related data leakage.

### 3.3. Proposed RGB Plantar Pressure Imaging Framework

Following preprocessing, the VGRF signals were transformed into RGB gait images using the proposed signal-to-image encoding framework. For each walking trial, the 16 sensor channels were organized into left- and right-foot matrices:

(3) $$L=\left[l_{t,s}\right]\in R^{T\times 8}$$

(4) $$R=\left[l_{t,s}\right]\in R^{T\times 8}$$

where t=1,…,T denotes the temporal sample index and s=1,…,8 denotes the ordered sensor index. Thus, the vertical axis represents successive temporal samples, whereas the horizontal axis represents the eight ordered plantar sensor channels. This representation should therefore be interpreted as a time–sensor signal matrix rather than an anatomical two-dimensional plantar pressure map.

To explicitly encode bilateral differences, an element-wise absolute difference matrix was calculated between corresponding left- and right-foot sensor channels:

(5) D=|L−R|

where $D\in R^{T\times 8}$. As described in the preprocessing section, L, R, and D were independently normalized to [0,1] within each walking trial. Each normalized matrix was subsequently converted to an 8-bit grayscale intensity matrix and resized from T×8 to the target image resolution H×H, where H∈{224,384,512}, using bilinear interpolation:

(6) $$L_{H}=B_{H}\left(\tilde{L}\right),\;R_{H}=B_{H}\left(\tilde{R}\right),\;D_{H}=B_{H}\left(\tilde{D}\right)$$

where $B_{H}\left(.\right)$ denotes bilinear resizing to H×H.

The three resized matrices were assigned to the red, green, and blue channels, respectively:

(7) $$I_{RGB}=stack(L_{H},R_{H},D_{H})\in R^{H\times H\times 3}$$

The red channel represents the temporal–sensor pattern of the left-foot VGRF signals, the green channel represents that of the right foot, and the blue channel represents the absolute bilateral difference between corresponding sensor signals. This encoding converts the multichannel VGRF time series into a standardized three-channel image representation compatible with conventional CNN- and transformer-based image classification architectures. Each complete gait recording was treated as a single sample and converted into one RGB image at each evaluated resolution. No temporal segmentation, overlapping windows, or gait-cycle extraction and normalization were performed. Because the recordings varied in duration, the resulting T×8 matrices contained different numbers of temporal samples. Rather than truncating or padding the recordings, each complete matrix was directly resized to the target resolution (224 × 224, 384 × 384, or 512 × 512) using bilinear interpolation. Segment length and overlap ratio were not applicable in the present study. Because some participants contributed multiple gait recordings, the number of images per participant corresponded to the number of valid recordings available for that participant.

### 3.4. Deep Learning Models

Four representative image-based deep learning architectures were evaluated using the proposed plantar pressure gait image representation: ResNet50, EfficientNet-B0, Vision Transformer (ViT-Tiny), and Swin Transformer (Swin-Tiny). ResNet50 and EfficientNet-B0 were selected as representative convolutional neural networks (CNNs), whereas ViT-Tiny and Swin-Tiny represented transformer-based architectures. ResNet50 uses residual connections to facilitate hierarchical feature learning, while EfficientNet-B0 employs balanced network scaling to improve computational efficiency. ViT-Tiny uses self-attention to model global relationships among image patches, whereas Swin-Tiny combines hierarchical feature extraction with shifted-window attention. The classification head of each architecture was configured for binary classification of Parkinson’s disease (PD) and healthy controls (HCs). The image-based models were initialized with ImageNet-pretrained weights when available and subsequently fine-tuned using the proposed gait images.

To provide direct signal-based reference baselines, a one-dimensional convolutional neural network (1D-CNN) and a bidirectional long short-term memory (BiLSTM) network were additionally implemented. These models directly processed the 16-channel VGRF signals without signal-to-image transformation. Each sensor channel was independently normalized using Min–Max normalization and linearly interpolated to 4096 time points, resulting in an input tensor of 16*4096. The 1D-CNN consisted of three convolutional layers with 64, 128, and 256 output channels and kernel sizes of 9, 7, and 5, respectively. Batch normalization and ReLU activation were applied after each convolutional layer, with max pooling after the first two layers, followed by adaptive average pooling, dropout (0.3), and a fully connected classification layer. The BiLSTM consisted of two bidirectional LSTM layers with a hidden size of 64 and an inter-layer dropout rate of 0.2. The sequence outputs were averaged across the temporal dimension and passed through dropout (0.3) and a fully connected classification layer. Unlike the image-based architectures, the 1D-CNN and BiLSTM were trained from random initialization because ImageNet-pretrained weights are not directly applicable to these one-dimensional signal architectures. Accordingly, these models were considered direct signal-based reference baselines rather than strictly initialization-matched counterparts to the image-based models.

Within the model-development portion of each cross-validation iteration, 15% of the participants were selected as the validation set using stratified random splitting based on the PD/control label. The validation split was performed at the subject level, ensuring that all recordings from the same participant remained within the same partition, and the same procedure was independently applied to each fold and repetition. Model selection and early stopping were based on validation AUC. A patience of 15 epochs was used; training was terminated when validation AUC failed to improve for 15 consecutive epochs, and the model state achieving the highest validation AUC was retained for evaluation on the corresponding held-out test fold.

All models were trained for a maximum of 80 epochs using the AdamW optimizer. To ensure a consistent training protocol, an initial learning rate of 5 × 10^−6^ and a weight decay of 1 × 10^−2^ were used for both the image-based models and the direct time-series baselines. For the image-based models, batch sizes of 16, 8, and 4 were used for input resolutions of 224 × 224, 384 × 384, and 512 × 512, respectively, whereas a batch size of 16 was used for the 1D-CNN and BiLSTM models. Class imbalance was addressed using weighted cross-entropy loss, with class weights calculated exclusively from the training-recording counts within each fold. Label smoothing of 0.05 was applied to the image-based models, whereas the 1D-CNN and BiLSTM used weighted cross-entropy without label smoothing. No geometric image augmentation, such as flipping or rotation, was applied to the image-based models because these transformations could alter the temporal–sensor organization of the encoded gait images. A base random seed of 40 was used for reproducibility. All models used a 15% subject-level validation subset for model selection based on validation AUC, with a maximum of 80 epochs and an early-stopping patience of 15 epochs. The detailed training configurations are summarized in Table 1.

Representation-level ablation experiments were conducted to quantify the contributions of the different components of the proposed gait representation. In addition to the complete RGB representation, four configurations were evaluated: RG, containing left- and right-foot information without an explicit bilateral-difference channel; B, containing only the absolute bilateral-difference information; R, containing only left-foot information; and G, containing only right-foot information. To isolate the effect of the representation while holding the model architecture and image resolution constant, all ablation experiments were conducted using Swin-Tiny at a fixed resolution of 384 × 384.

All experiments were conducted on a workstation equipped with an Intel Core i9-13900K processor, 128 GB RAM, and an NVIDIA GeForce RTX 4090 GPU.

#### 3.4.1. ResNet50

ResNet50 is one of the most widely adopted convolutional neural networks for image recognition [26]. It employs residual learning through shortcut connections, allowing gradients to propagate efficiently during backpropagation and alleviating the degradation problem encountered in deep neural networks. The residual architecture enables the extraction of hierarchical image features while maintaining stable optimization, making ResNet50 a strong baseline for medical image classification.

#### 3.4.2. EfficientNet-B0

EfficientNet-B0 adopts a compound scaling strategy that simultaneously scales network depth, width, and input resolution [27]. Compared with conventional CNN architectures, EfficientNet-B0 achieves superior accuracy while requiring substantially fewer parameters and computational resources. Its lightweight design makes it particularly suitable for intelligent healthcare systems where computational efficiency is an important consideration.

#### 3.4.3. Vision Transformer (ViT)

Unlike CNNs that primarily capture local receptive fields, Vision Transformer (ViT) divides the input image into fixed-size patches and employs self-attention mechanisms to learn global contextual relationships among image regions [28]. This architecture enables the model to capture long-range dependencies and complex spatial interactions, making it effective for learning discriminative gait representations from RGB plantar pressure images.

#### 3.4.4. Swin Transformer

Swin Transformer introduces a hierarchical architecture with shifted window-based multi-head self-attention [29]. Compared with the original Vision Transformer, Swin Transformer significantly reduces computational complexity while preserving both local and global contextual information. The shifted-window mechanism enables efficient feature aggregation across neighboring image regions and has demonstrated excellent performance in numerous medical image classification applications.

### 3.5. Dataset Partitioning

To ensure a robust evaluation of model performance while preventing subject-level data leakage, repeated stratified subject-level five-fold cross-validation with three repetitions was performed, resulting in 15 evaluation runs. The final analytical cohort consisted of 165 participants (93 patients with Parkinson’s disease [PD] and 72 healthy controls [HCs]), contributing 306 gait recordings. Stratification and data partitioning were performed at the participant level, such that all recordings from the same participant were assigned to the same fold within each repetition. The same outer cross-validation partitions were used across the evaluated models, image resolutions, and representation experiments to facilitate consistent comparisons. The detailed validation-set selection and early-stopping procedures are described in the model training section. Because some participants contributed multiple gait recordings and the cross-validation procedure was repeated three times, the pooled out-of-fold predictions were not statistically independent observations at the participant level. Each of the 306 gait recordings was evaluated once per repetition, resulting in 918 pooled recording-level predictions across the three repetitions. These predictions represent repeated recording-level model evaluations and should not be interpreted as 918 independent participants.

### 3.6. Performance Evaluation

The performance of the evaluated deep learning models was comprehensively assessed using multiple classification metrics to account for both overall predictive performance and class imbalance. Model performance was evaluated on the held-out test subjects in each fold of the repeated stratified subject-level cross-validation procedure. The evaluation metrics included Accuracy, Precision, Recall (Sensitivity), Specificity, F1-score, and the Area Under the Receiver Operating Characteristic Curve (AUC). Given the unequal distribution of patients with Parkinson’s disease and healthy controls, Balanced Accuracy and the Matthews Correlation Coefficient (MCC) were additionally included to provide a more robust assessment of classification performance across both classes.

For each model configuration, performance metrics were calculated for each of the five folds and repeated across three cross-validation repetitions, resulting in 15 evaluation runs. Performance across these runs was summarized as the mean and standard deviation to characterize variability across different subject partitions. Because the same participants contributed to multiple repetitions of the cross-validation procedure, the 15 fold-level estimates were not considered statistically independent observations and were therefore used for descriptive performance summarization rather than primary statistical inference.

For participant-level evaluation, out-of-fold predicted probabilities were aggregated for each participant. For participants with multiple gait recordings, prediction probabilities were first averaged across the available recordings within each cross-validation repetition and then averaged across the three repetitions, resulting in one aggregated predicted probability per participant. Performance metrics were subsequently calculated at the participant level. To account for uncertainty without treating repeated fold-level estimates as independent observations, 95% confidence intervals were estimated using 2000 bootstrap resamples at the participant level. For pairwise model comparisons, paired participant-level bootstrap resampling was used to estimate the confidence intervals of between-model performance differences.

## 4. Results

### 4.1. Raw Time-Series vs. RGB Image-Based Representation

To evaluate the signal-to-image representation relative to direct time-series learning, the proposed image-based approach was compared with two reference models that directly processed the 16-channel VGRF time series, including a 1D-CNN and a BiLSTM. All models were evaluated using the same repeated stratified subject-level five-fold cross-validation protocol with three repetitions. As shown in Table 2, the BiLSTM achieved a mean accuracy of 51.67 ± 21.38%, F1-score of 39.58 ± 42.81%, AUC of 65.26 ± 19.02%, balanced accuracy of 51.32 ± 4.59%, and MCC of 0.036 ± 0.106. The large standard deviations across several metrics indicate substantial variability in BiLSTM performance across subject partitions. The 1D-CNN showed more stable performance, achieving a mean accuracy of 68.75 ± 12.58%, F1-score of 73.05 ± 13.35%, AUC of 80.24 ± 10.46%, balanced accuracy of 71.28 ± 9.20%, and MCC of 0.412 ± 0.176. Among the evaluated complete RGB configurations, ViT-Tiny at 384 × 384 achieved a mean accuracy of 77.64 ± 6.57%, F1-score of 82.82 ± 6.24%, AUC of 86.40 ± 5.79%, balanced accuracy of 76.84 ± 6.36%, and MCC of 0.527 ± 0.120. Compared with the 1D-CNN baseline, ViT-Tiny showed numerically higher mean accuracy by 8.89 percentage points, F1-score by 9.77 percentage points, and AUC by 6.16 percentage points. These differences are presented descriptively and should not be interpreted as evidence that image-based representations are generally superior to direct time-series approaches.

### 4.2. RGB Image-Based CNN vs. Transformer Comparison

Table 3 summarizes the performance of the four image-based architectures using the complete RGB representation at input resolutions of 224 × 224, 384 × 384, and 512 × 512. Results are reported as the mean ± standard deviation across 15 runs from repeated stratified subject-level five-fold cross-validation with three repetitions. At 224 × 224, Swin-Tiny achieved the highest mean accuracy (73.93 ± 7.52%), F1-score (79.87 ± 7.11%), and AUC (82.53 ± 7.20%), followed closely by ViT-Tiny. ResNet50 and EfficientNet-B0 showed lower discrimination, with mean AUCs of 58.52 ± 9.86% and 55.66 ± 10.10%, respectively. At 384 × 384, ViT-Tiny achieved the highest mean accuracy (77.64 ± 6.57%), F1-score (82.82 ± 6.24%), AUC (86.40 ± 5.79%), balanced accuracy (76.84 ± 6.36%), and MCC (0.527 ± 0.120). Swin-Tiny achieved a mean accuracy of 75.34 ± 8.33% and AUC of 83.96 ± 7.50%, with the highest precision (87.54 ± 8.27%) and specificity (75.92 ± 14.72%) at this resolution. At 512 × 512, ViT-Tiny achieved the highest mean accuracy (78.04 ± 6.53%) and F1-score (83.39 ± 5.78%) among the complete RGB configurations, with an AUC of 86.10 ± 7.34%. Increasing the resolution did not consistently improve performance across architectures. Notably, ResNet50 at 512 × 512 exhibited high sensitivity (85.28 ± 30.17%) but markedly low specificity (25.31 ± 31.67%), indicating a strong tendency to classify samples as PD and poor discrimination of healthy controls. The large standard deviations further indicate substantial variability across the repeated cross-validation folds.

### 4.3. Ablation Analysis of RGB Channel Representations

To investigate the contribution of individual components of the proposed plantar pressure representation while controlling for model architecture and input resolution, an ablation analysis was performed using Swin-Tiny at 384 × 384 resolution. Five encoding configurations were evaluated under the same repeated stratified subject-level five-fold cross-validation protocol: left-foot pressure only (R), right-foot pressure only (G), combined left- and right-foot pressure (RG), the complete RGB representation incorporating left-foot, right-foot, and absolute bilateral-difference information (RGB), and absolute bilateral-difference information only (B). As shown in Table 4, the unilateral representations yielded lower mean performance than the bilateral representations. The R representation achieved a mean accuracy of 68.62 ± 6.84% and AUC of 77.07 ± 6.80%, whereas the G representation achieved 68.49 ± 10.16% and 76.35 ± 9.95%, respectively. Combining left- and right-foot information (RG) increased the mean accuracy to 72.73 ± 8.44%, F1-score to 78.64 ± 8.99%, AUC to 81.73 ± 8.11%, and balanced accuracy to 72.18 ± 4.74%. The complete RGB representation further increased the mean accuracy to 75.34 ± 8.33%, F1-score to 80.37 ± 8.29%, AUC to 83.96 ± 7.50%, balanced accuracy to 76.11 ± 6.22%, and MCC to 0.501 ± 0.118. Among the five configurations, the B representation achieved the highest mean accuracy (79.42 ± 7.13%), F1-score (84.39 ± 6.34%), AUC (86.68 ± 6.56%), and MCC (0.544 ± 0.147), with a balanced accuracy of 77.26 ± 7.80%. Compared with the complete RGB representation under the same Swin-Tiny architecture and 384 × 384 resolution, the B representation showed numerically higher mean accuracy by 4.08 percentage points and mean AUC by 2.72 percentage points.

### 4.4. ROC Curve Analysis

**Figure 2:**
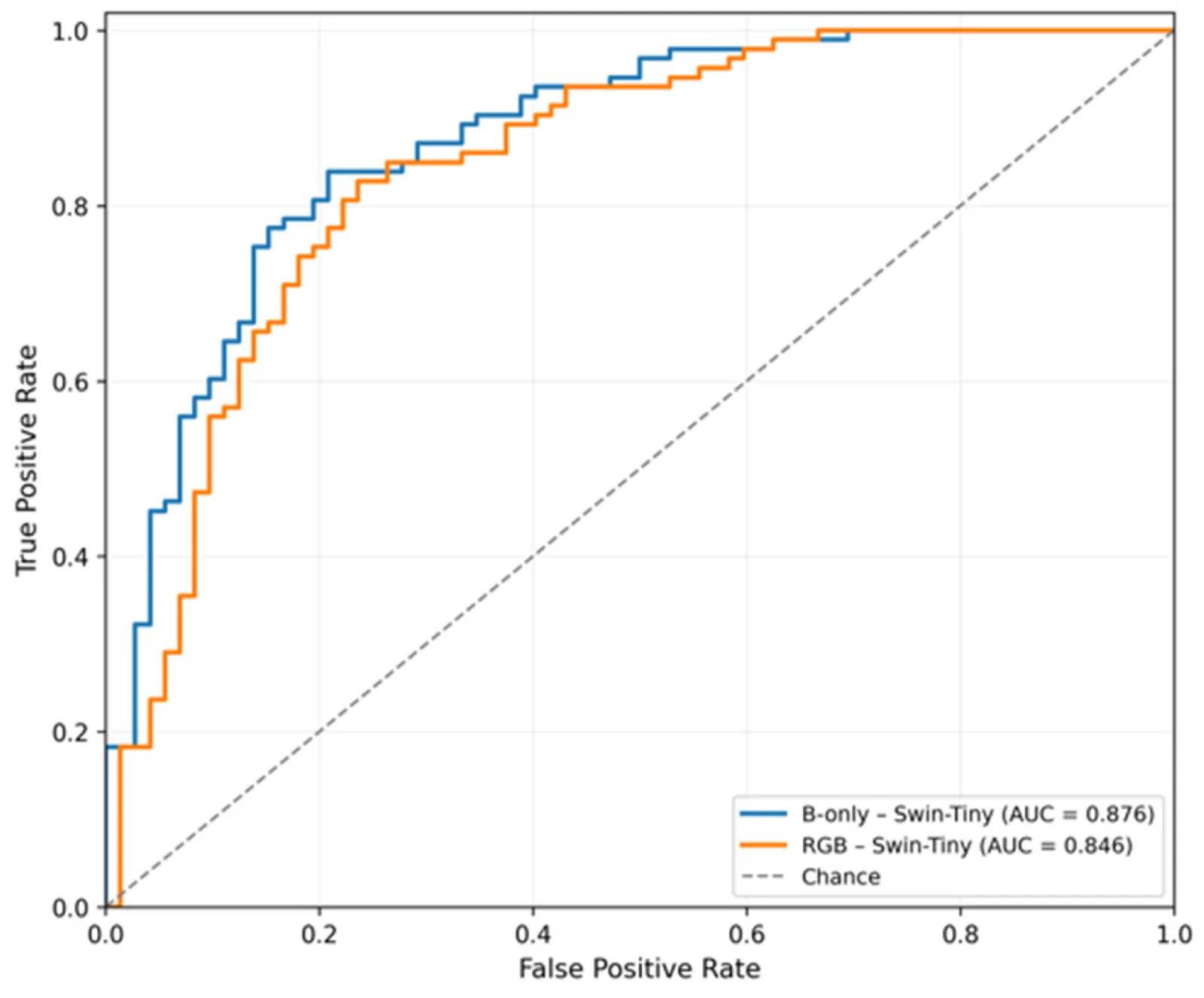
Participant-level receiver operating characteristic curves for the complete RGB and B-only representations using Swin-Tiny at an input resolution of 384 × 384. ROC curves were generated from aggregated participant-level out-of-fold predictions (*N* = 165). B denotes the absolute bilateral difference |L − R|.

Figure 2 presents the receiver operating characteristic (ROC) curves based on aggregated participant-level out-of-fold predictions for the complete RGB and B-only representations using Swin-Tiny at an input resolution of 384 × 384. For participants with multiple gait recordings, predicted probabilities were first averaged across recordings within each cross-validation repetition and subsequently averaged across the three repetitions, resulting in one aggregated prediction probability per participant (*N* = 165). The complete RGB representation, which incorporates left-foot, right-foot, and absolute bilateral-difference information, achieved a participant-level AUC of 0.846, whereas the B-only representation, representing the absolute bilateral difference |L − R|, achieved an AUC of 0.876. The two ROC curves showed broadly similar discrimination patterns across classification thresholds, with the B-only representation yielding a numerically higher AUC. However, the paired participant-level bootstrap comparison showed a ΔAUC of 0.030 (95% CI: −0.011 to 0.068). Because the confidence interval included zero, the observed difference was not interpreted as evidence of statistical superiority.

### 4.5. Confusion Matrix and Class-Specific Analysis

**Figure 3:**
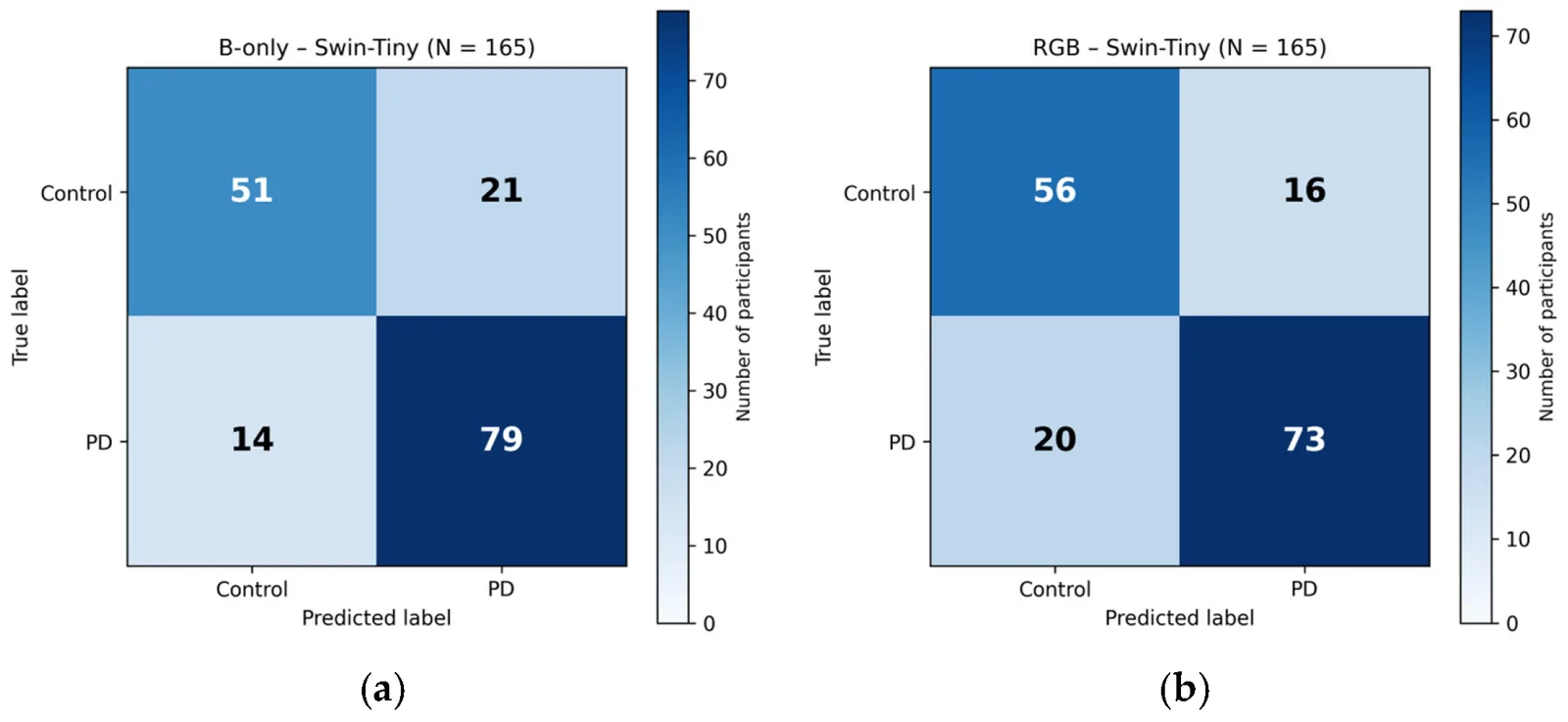
Participant-level confusion matrices for (**a**) the B-only representation and (**b**) the complete RGB representation using Swin-Tiny at an input resolution of 384 × 384. Confusion matrices were calculated from aggregated participant-level out-of-fold predictions (*N* = 165) using a classification threshold of 0.5. The B-only representation denotes the absolute bilateral difference, |L − R|. Control and PD denote healthy controls and participants with Parkinson’s disease, respectively.

Figure 3 presents the participant-level confusion matrices for the B-only and complete RGB representations using the same Swin-Tiny architecture at an input resolution of 384 × 384. The confusion matrices were generated from aggregated participant-level out-of-fold predictions for 165 participants using a classification threshold of 0.5. As shown in Figure 3a, the B-only representation correctly classified 79 of 93 participants with PD and 51 of 72 healthy controls, while 14 participants with PD were misclassified as controls and 21 healthy controls were misclassified as PD. This corresponded to a sensitivity of 84.95%, specificity of 70.83%, precision of 79.00%, F1-score of 81.87%, and balanced accuracy of 77.89%. For the complete RGB representation (Figure 3b), 73 of 93 participants with PD and 56 of 72 healthy controls were correctly classified, whereas 20 participants with PD were misclassified as controls and 16 healthy controls were misclassified as PD. The corresponding sensitivity, specificity, precision, F1-score, and balanced accuracy were 78.49%, 77.78%, 82.02%, 80.22%, and 78.14%. The two representations exhibited different class-specific error patterns. The B-only representation showed numerically higher sensitivity (84.95% vs. 78.49%) and fewer false-negative PD classifications (14 vs. 20), whereas the complete RGB representation showed higher specificity (77.78% vs. 70.83%) and fewer false-positive classifications among healthy controls (16 vs. 21). Balanced accuracy was similar between the two representations (77.89% vs. 78.14%). These differences are interpreted descriptively rather than as evidence of superiority of either representation.

### 4.6. Statistical Comparison Between Models

To account for the dependence among fold-level estimates generated by repeated cross-validation, statistical comparisons were performed using aggregated participant-level out-of-fold predictions rather than treating the 15 cross-validation runs as independent observations. For participants with multiple gait recordings, predicted probabilities were first averaged across recordings within each cross-validation repetition and subsequently averaged across the three repetitions, resulting in one aggregated prediction probability for each of the 165 participants. Pairwise differences between selected configurations were evaluated using 2000 paired participant-level bootstrap resamples, with 95% confidence intervals (CIs) calculated for the differences in AUC, as shown in Table 5. The B-only Swin-Tiny configuration at 384 × 384 achieved a participant-level AUC of 0.876, compared with 0.846 for the complete RGB Swin-Tiny configuration, corresponding to a ΔAUC of 0.030 (95% CI: −0.011 to 0.068). RGB ViT-Tiny at 384 × 384 achieved an AUC of 0.872, compared with 0.846 for RGB Swin-Tiny at the same resolution (ΔAUC = 0.026, 95% CI: −0.025 to 0.075). The difference between B-only Swin-Tiny and RGB ViT-Tiny at 384 × 384 was small (ΔAUC = 0.003, 95% CI: −0.049 to 0.062). Increasing the ViT-Tiny input resolution from 384 × 384 to 512 × 512 also did not improve participant-level AUC, with a ΔAUC of −0.014 (95% CI: −0.053 to 0.023). All 95% CIs for the paired AUC differences included zero. Although numerical differences were observed among the evaluated configurations, the participant-level bootstrap analysis did not provide evidence of statistical superiority for any of these selected comparisons. These differences were therefore interpreted descriptively.

### 4.7. Computational Efficiency

The computational efficiency of the evaluated deep learning architectures was assessed in terms of the number of trainable parameters, training time, and inference time per sample. As shown in Table 6, substantial differences in computational complexity were observed across model architectures and input resolutions. EfficientNet-B0 contained the fewest trainable parameters (4.01 million), followed by ViT-Tiny (approximately 5.52–5.68 million), whereas ResNet50 and Swin-Tiny contained 23.51 million and 27.52 million parameters, respectively. Among the RGB image-based models, ViT-Tiny demonstrated a favorable balance between predictive performance and computational requirements. At an input resolution of 384 × 384, ViT-Tiny contained 5.60 million trainable parameters, with a mean training time of 1372.33 ± 569.86 s and an average inference time of 209.01 ms per sample. In comparison, Swin-Tiny at the same resolution required 27.52 million parameters and a mean training time of 2015.08 ± 713.91 s, with an inference time of 212.19 ms per sample. Thus, ViT-Tiny required approximately 80% fewer trainable parameters and a shorter mean training time while achieving the strongest overall RGB-based discrimination performance. Input resolution also influenced computational requirements. For most CNN architectures, increasing the image resolution resulted in longer training and inference times. For example, ResNet50 inference time increased from 176.92 ms at 224 × 224 to 253.69 ms at 512 × 512, while EfficientNet-B0 increased from 174.56 to 233.89 ms. A similar increase in inference time was observed for ViT-Tiny, from 172.10 ms at 224 × 224 to 231.19 ms at 512 × 512. These findings indicate that increasing image resolution imposes additional computational costs without consistently producing corresponding improvements in classification performance. For the asymmetry-only representation, Swin-Tiny at 384 × 384 required 27.52 million parameters, with a mean training time of 1977.63 ± 754.16 s and an inference time of 209.14 ms per sample. Although this configuration achieved the highest mean performance in the representation ablation analysis, its substantially larger parameter count compared with ViT-Tiny highlights a trade-off between predictive performance and model complexity. Overall, the results suggest that ViT-Tiny at 384 × 384 provides a computationally efficient RGB-based configuration, whereas the asymmetry-based Swin model offers slightly higher mean discrimination at the cost of greater model complexity.

## 5. Discussion

This study developed and systematically evaluated a signal-to-image deep learning framework for distinguishing participants with diagnosed Parkinson’s disease (PD) from healthy controls using plantar pressure gait signals. Repeated stratified subject-level five-fold cross-validation with three repetitions was used to reduce dependence on a single train–test partition. The results showed that the proposed image representation provided competitive classification performance relative to the evaluated direct time-series baselines. Among the complete RGB configurations, ViT-Tiny showed strong overall performance, whereas the representation ablation using Swin-Tiny at 384 × 384 indicated that the B-only representation based on the absolute bilateral difference |L − R| achieved the highest mean AUC within that specific ablation setting. A central contribution of this study is the transformation of multidimensional plantar pressure signals into an image-compatible representation that can be processed using established computer vision architectures. The complete RGB representation encodes left-foot pressure, right-foot pressure, and the absolute bilateral difference in separate channels. This approach avoids manual extraction of predefined gait descriptors and provides a common representation for comparing CNN- and transformer-based architectures. The contribution of the present work lies primarily in the representation and evaluation framework rather than in proposing a new neural network architecture.

Comparison with direct time-series models showed that the RGB-based ViT-Tiny at 384 × 384 achieved higher mean accuracy, F1-score, and AUC than the evaluated 1D-CNN and BiLSTM baselines. These findings should not be interpreted as demonstrating the general superiority of image-based representations over time-series learning. The BiLSTM exhibited substantial variability across cross-validation folds, and exhaustive architecture-specific hyperparameter optimization was not performed. The results therefore indicate that the proposed signal-derived image representation is a competitive alternative within the models and training settings evaluated in this study. The representation ablation provides additional insight into the contribution of bilateral information. Under the fixed Swin-Tiny architecture at 384 × 384, the left-only and right-only representations achieved mean AUCs of 77.07% and 76.35%, respectively, whereas combining both feet increased the mean AUC to 81.73%. The complete RGB representation achieved a mean AUC of 83.96%, and the B-only representation based on |L − R| achieved the highest mean AUC of 86.68%. Participant-level analysis showed that the difference between B-only and RGB Swin-Tiny was not statistically conclusive (ΔAUC = 0.030, 95% CI: −0.011 to 0.068). The participant-level confusion matrices further showed different error profiles: B-only Swin-Tiny had higher sensitivity (84.95% vs. 78.49%), whereas RGB Swin-Tiny had higher specificity (77.78% vs. 70.83%). Their balanced accuracy was similar (77.89% vs. 78.14%). These findings suggest that the absolute bilateral-difference representation contains useful discriminative information, but they do not establish its superiority over the complete RGB representation. The ablation findings should also be interpreted within the scope of the experimental design. Representation comparisons were controlled by fixing the architecture and resolution to Swin-Tiny at 384 × 384; therefore, differences among R, G, RG, RGB, and B can be interpreted without simultaneously changing the model architecture. The ablation was conducted using only one architecture and one resolution. Whether the same representation effects occur with ViT, CNN-based architectures, or other input resolutions remain unknown and should be investigated in future studies. Image resolution influenced model performance, but increasing resolution did not consistently improve classification. Among the complete RGB configurations, ViT-Tiny performed strongly at both 384 × 384 and 512 × 512, whereas higher resolution was not consistently beneficial for the CNN architectures. Notably, ResNet50 at 512 × 512 exhibited a pronounced class-specific prediction bias, with high sensitivity (85.28%) but markedly low specificity (25.31%), indicating a strong tendency to predict the PD class. The large standard deviations for sensitivity and specificity further indicate substantial variability across cross-validation folds. This finding highlights the importance of considering specificity, balanced accuracy, and MCC alongside sensitivity and overall accuracy when evaluating classification performance. Because these images were generated by resizing underlying sensor matrices rather than acquired as natural images, increasing image dimensions may introduce interpolation redundancy without necessarily adding new discriminative information. Resolution should therefore be considered together with model architecture, computational cost, and representation design.

The participant-level analysis provides a more conservative interpretation of model differences. Because the 15 estimates generated by repeated five-fold cross-validation are correlated, they were retained as descriptive measures of variability rather than treated as independent observations for statistical inference. Out-of-fold probabilities were instead aggregated to one prediction per participant, and participant-level bootstrap resampling was used to estimate confidence intervals and paired performance differences. The resulting confidence intervals for selected comparisons included zero, emphasizing that small numerical differences between the highest-performing configurations should not be interpreted as evidence of statistical superiority. The clinical implications of these findings should remain conservative. The present study evaluated classification between participants with established PD and healthy controls, rather than prospective diagnosis, screening, or early disease detection. The PD cohort consisted of participants with diagnosed disease at Hoehn and Yahr stages 2–3; the current results do not provide evidence for identifying individuals with prodromal or early-stage PD. Although Hoehn and Yahr stage and UPDRS measures are available for characterizing disease severity, the present study was not designed to determine whether the proposed representation predicts clinical severity or detects PD before conventional diagnosis. Future studies should explicitly investigate associations between model-derived patterns and H&Y or UPDRS measures and include earlier-stage, prodromal, and clinically relevant differential-diagnosis populations before claims regarding early detection can be considered.

Several additional limitations should be acknowledged. The study used a single publicly available dataset collected under a specific experimental protocol. Internal repeated cross-validation improves the robustness of performance estimation but cannot substitute for external validation. The observed specificity remained moderate, including participant-level specificities of 70.83% and 77.78% for the B-only and RGB Swin-Tiny configurations, respectively. These results are insufficient to support claims of diagnostic or clinical decision-support performance. Gait characteristics may be influenced by potential confounding factors including age, sex, medication status, disease severity, and data-collection protocol. These factors were not comprehensively controlled in the present analysis and may contribute to the observed classification patterns. Future studies should examine these variables explicitly through stratified or adjusted analyses and validate the framework across independent institutions, sensor systems, and acquisition protocols. Per-trial Min–Max normalization removes absolute between-trial differences in loading magnitude, and the representation ablation was restricted to one architecture and resolution.

Future work should prioritize external and multicenter validation rather than immediate clinical deployment. Larger and more heterogeneous cohorts could be used to evaluate the robustness of the proposed representation across demographic characteristics, medication states, disease stages, and acquisition environments. Associations between the absolute bilateral-difference representation and established clinical measures such as H&Y stage and UPDRS should also be investigated. Multimodal integration with inertial sensors, video-based gait analysis, or clinical variables may provide complementary information. These investigations will be necessary to determine whether the proposed framework can progress from discrimination between diagnosed PD and healthy controls toward more clinically informative gait assessment.

## 6. Conclusions

This study developed a signal-to-image deep learning framework for classifying participants with diagnosed Parkinson’s disease (PD) and healthy controls using plantar pressure gait signals. Bilateral VGRF signals were transformed into RGB images encoding left-foot pressure, right-foot pressure, and the absolute bilateral difference, and were evaluated using repeated stratified subject-level cross-validation. Among the complete RGB configurations, ViT-Tiny at 384 × 384 achieved a favorable balance between classification performance and computational complexity, with a mean accuracy of 77.64%, F1-score of 82.82%, and AUC of 86.40%. The image-based approach also achieved competitive performance relative to the evaluated direct time-series baselines. Representation ablation using Swin-Tiny at 384 × 384 showed that the B-only representation based on the absolute bilateral difference achieved the highest mean AUC (86.68%) among the five evaluated encodings. However, participant-level bootstrap analysis did not provide evidence of statistical superiority over the complete RGB representation. Overall, these findings demonstrate the feasibility of signal-derived gait-image representations for PD classification and highlight the potential discriminative value of bilateral gait differences. Given the single-dataset design, moderate specificity, and potential demographic, clinical, and acquisition-related confounders, further validation in independent and more heterogeneous cohorts is required before considering diagnostic or clinical application.

## Figures and Tables

**Table 1:** Training configuration for all deep learning models.

Parameter	Image-Based Models	1D-CNN/BiLSTM
Input	RGB images (224, 384, 512)	16-channel VGRF (4096 points)
Initialization	ImageNet pretrained	Random
Optimizer	AdamW	AdamW
Learning rate	5 × 10^−6^	5 × 10^−6^
Weight decay	1 × 10^−2^	1 × 10^−2^
Batch size	16/8/4	16
Maximum epochs	80	80
Early stopping	15	15
Selection metric	Validation AUC	Validation AUC
Loss	Weighted cross-entropy + label smoothing (0.05)	Weighted cross-entropy
Validation set	15% of development subjects	15% of development subjects
Random seed	40	40

**Table 2:** Comparison of direct time-series and RGB image-based deep learning approaches using repeated subject-level cross-validation.

Representation	Model	Accuracy (%)	Precision (%)	Recall (%)	Specificity (%)	F1-Score (%)	AUC (%)	Balanced Accuracy (%)	MCC
VGRF time series	BiLSTM	51.67 ± 21.38	40.76 ± 40.14	46.17 ± 50.63	56.47 ± 49.56	39.58 ± 42.81	65.26 ± 19.02	51.32 ± 4.59	0.036 ± 0.106
VGRF time series	1D-CNN	68.75 ± 12.58	86.58 ± 10.82	66.59 ± 19.77	75.97 ± 18.68	73.05 ± 13.35	80.24 ± 10.46	71.28 ± 9.20	0.412 ± 0.176
RGB gait image	ViT-Tiny (384 × 384)	77.64 ± 6.57	86.98 ± 8.78	80.56 ± 11.63	73.12 ± 17.02	82.82 ± 6.24	86.40 ± 5.79	76.84 ± 6.36	0.527 ± 0.120

**Table 3:** Classification performance of CNN- and transformer-based models using RGB plantar pressure gait representations across different input resolutions.

Resolution	Model	Accuracy (%)	Precision (%)	Recall (%)	Specificity (%)	F1-Score (%)	AUC (%)	Balanced Acc. (%)	MCC
224 × 224	ResNet50	54.69 ± 11.31	72.92 ± 9.71	52.48 ± 18.91	58.88 ± 16.64	59.51 ± 16.47	58.52 ± 9.86	55.68 ± 8.04	0.104 ± 0.159
	EfficientNet-B0	53.71 ± 8.50	70.09 ± 7.39	56.19 ± 17.04	46.94 ± 19.04	61.15 ± 12.41	55.66 ± 10.10	51.57 ± 5.95	0.023 ± 0.111
	ViT-Tiny	72.33 ± 10.36	83.21 ± 9.28	75.71 ± 15.36	67.49 ± 14.01	78.21 ± 10.50	82.32 ± 8.39	71.60 ± 8.14	0.421 ± 0.158
	Swin-Tiny	73.93 ± 7.52	85.07 ± 9.42	77.58 ± 14.20	69.15 ± 18.62	79.87 ± 7.11	82.53 ± 7.20	73.37 ± 6.53	0.461 ± 0.122
384 × 384	ResNet50	62.04 ± 13.11	77.98 ± 8.91	65.42 ± 25.94	53.17 ± 24.94	66.61 ± 22.59	60.45 ± 14.93	59.30 ± 9.29	0.194 ± 0.182
	EfficientNet-B0	59.88 ± 8.77	77.65 ± 6.81	58.92 ± 13.41	62.10 ± 13.57	66.08 ± 11.09	63.01 ± 11.07	60.51 ± 7.13	0.197 ± 0.138
	ViT-Tiny	77.64 ± 6.57	86.98 ± 8.78	80.56 ± 11.63	73.12 ± 17.02	82.82 ± 6.24	86.40 ± 5.79	76.84 ± 6.36	0.527 ± 0.120
	Swin-Tiny	75.34 ± 8.33	87.54 ± 8.27	76.31 ± 13.92	75.92 ± 14.72	80.37 ± 8.29	83.96 ± 7.50	76.11 ± 6.22	0.501 ± 0.118
512 × 512	ResNet50	66.53 ± 14.42	73.07 ± 8.75	85.28 ± 30.17	25.31 ± 31.67	73.77 ± 22.54	67.17 ± 14.66	55.30 ± 7.63	0.165 ± 0.216
	EfficientNet-B0	60.11 ± 10.23	76.19 ± 10.75	59.66 ± 14.59	60.02 ± 12.82	66.28 ± 13.23	63.53 ± 11.18	59.84 ± 9.03	0.182 ± 0.170
	ViT-Tiny	78.04 ± 6.53	86.60 ± 7.21	81.30 ± 9.76	71.64 ± 14.63	83.39 ± 5.78	86.10 ± 7.34	76.47 ± 7.09	0.516 ± 0.134
	Swin-Tiny	72.24 ± 15.08	83.71 ± 5.64	75.53 ± 23.60	66.93 ± 17.26	76.60 ± 19.09	82.46 ± 7.45	71.23 ± 8.29	0.435 ± 0.164

**Table 4:** Ablation analysis of plantar pressure encoding configurations using Swin-Tiny at 384 × 384 resolution for Parkinson’s disease classification.

Encoding	Model	Accuracy (%)	Precision (%)	Recall (%)	Specificity (%)	F1-Score (%)	AUC (%)	Balanced Acc. (%)	MCC
R	Left-foot pressure only	68.62 ± 6.84	83.58 ± 7.76	69.04 ± 13.19	67.88 ± 17.81	74.53 ± 7.87	77.07 ± 6.80	68.46 ± 6.58	0.352 ± 0.116
G	Right-foot pressure only	68.49 ± 10.16	82.26 ± 9.68	71.21 ± 16.99	65.05 ± 18.50	74.68 ± 10.70	76.35 ± 9.95	68.13 ± 8.10	0.356 ± 0.155
RG	Left- and right-foot pressure	72.73 ± 8.44	84.34 ± 8.46	76.86 ± 16.80	67.51 ± 16.70	78.64 ± 8.99	81.73 ± 8.11	72.18 ± 4.74	0.441 ± 0.099
RGB	Left + right + pressure asymmetry	75.34 ± 8.33	87.54 ± 8.27	76.31 ± 13.92	75.92 ± 14.72	80.37 ± 8.29	83.96 ± 7.50	76.11 ± 6.22	0.501 ± 0.118
B	Bilateral pressure asymmetry only	79.42 ± 7.13	87.13 ± 7.91	83.04 ± 10.98	71.48 ± 17.54	84.39 ± 6.34	86.68 ± 6.56	77.26 ± 7.80	0.544 ± 0.147

**Table 5:** Participant-level statistical comparison of selected deep learning models and plantar pressure representations using paired bootstrap analysis.

Comparison	AUC A	AUC B	ΔAUC (A − B)	95% CI
B-Swin384 vs. RGB-Swin384	0.876	0.846	+0.030	−0.011 to 0.068
RGB-ViT384 vs. RGB-Swin384	0.872	0.846	+0.026	−0.025 to 0.075
B-Swin384 vs. RGB-ViT384	0.876	0.872	+0.003	−0.049 to 0.062
RGB-ViT512 vs. RGB-ViT384	0.858	0.872	−0.014	−0.053 to 0.023

Note: ΔAUC represents AUC of the first configuration minus that of the second configuration. The 95% CIs were estimated using 2000 paired participant-level bootstrap resamples. All confidence intervals included zero.

**Table 6:** Computational efficiency of CNN- and transformer-based models using RGB plantar pressure gait images.

Model	Resolution	Parameters (M)	Training Time (s)	Inference Time (ms/Sample)
ResNet50	224 × 224	23.51	1625.95 ± 1078.47	176.92 ± 10.77
	384 × 384	23.51	2153.14 ± 1235.50	200.38 ± 22.83
	512 × 512	23.51	2698.62 ± 1232.37	253.69 ± 11.81
EfficientNet-B0	224 × 224	4.01	1446.50 ± 934.89	174.56 ± 7.25
	384 × 384	4.01	2021.07 ± 885.97	207.09 ± 11.18
	512 × 512	4.01	2153.62 ± 1001.64	233.89 ± 29.56
ViT-Tiny	224 × 224	5.52	1257.95 ± 323.85	172.10 ± 7.89
	384 × 384	5.60	1372.33 ± 569.86	209.01 ± 15.08
	512 × 512	5.68	1333.69 ± 281.85	231.19 ± 11.02
Swin-Tiny	224 × 224	27.52	1843.02 ± 616.03	175.90 ± 23.53
	384 × 384	27.52	2015.08 ± 713.91	212.19 ± 9.85
	512 × 512	27.52	1922.87 ± 656.75	247.77 ± 13.28

## Data Availability

The data used in this study are publicly available from the PhysioNet Parkinson’s Disease Gait Database. The dataset can be accessed at: https://physionet.org/content/gaitpdb/1.0.0/ (accessed on 1 September 2025). All data analyzed during this study are publicly available from the corresponding repository.
